# Oncostatin M receptor is a novel therapeutic target in cervical squamous cell carcinoma

**DOI:** 10.1002/path.4305

**Published:** 2014-02-08

**Authors:** Maria M Caffarel, Nicholas Coleman

**Affiliations:** 1 Department of Pathology, University of CambridgeUK

**Keywords:** oncostatin M receptor, squamous cell carcinoma, cervix, cell migration, invasion, angiogenesis, 5p gain

## Abstract

Cervical carcinoma is the second most common cause of cancer deaths in women worldwide. Treatments have not changed for decades and survival rates for advanced disease remain low. An exciting new molecular target for the treatment of cervical squamous cell carcinoma (SCC), and possibly for SCCs at other anatomical sites, is the oncostatin M receptor (OSMR). This cell surface cytokine receptor is commonly copy number gained and overexpressed in advanced cervical SCC, changes that are associated with significantly worse clinical outcomes. OSMR overexpression in cervical SCC cells results in enhanced responsiveness to the major ligand oncostatin M (OSM), which induces several pro-malignant effects, including a pro-angiogenic phenotype and increased cell migration and invasiveness. OSMR is a strong candidate for antibody-mediated inhibition, a strategy that has had a major impact on haematological malignancies and various solid tumours such as HER2-positive breast cancers. © 2013 The Authors. *The Journal of Pathology* published by John Wiley & Sons Ltd on behalf of Pathological Society of Great Britain and Ireland.

## Introduction

Despite a reduction in incidence and mortality rates since the introduction of population-wide screening programmes in developed countries, cervical carcinoma remains the second most common cause of cancer deaths in women worldwide 1. Each year there are ∼500 000 new cases and ∼270 000 deaths from cervical carcinoma. The global burden of the disease is expected to be reduced long term (over 20–30 years), due to the implementation of vaccination programmes to prevent infection with the main causative agent, high-risk human papillomavirus (HR-HPV). However, worldwide vaccine coverage is still very low and current vaccines target a restricted range of HR-HPV types. Current treatments for cervical carcinoma have not changed for decades and survival rates for advanced disease remain poor. Therefore, new therapeutic strategies for disease management are urgently needed 2.

Most cervical carcinomas are squamous cell carcinomas (SCCs), which arise from precursor squamous intraepithelial lesions (SILs). While infection with HR-HPV is necessary for cervical carcinogenesis, it is not sufficient for progression to malignancy. Low-grade SILs often regress and only a minority of women infected with HPV develop cervical carcinoma, with malignant progression involving a long latent period where additional ‘hits’ are needed 3. An important feature of cervical carcinogenesis is genomic instability caused by deregulated expression of HR-HPV oncogenes in proliferating epithelial cells 4. These changes lead to the selection of particular genomic copy number imbalances, which are likely to provide a selective advantage through altered expression of ‘driver’ host genes.

## Chromosome 5p is frequently copy number gained and amplified in squamous cell carcinoma

Several groups have performed integrative genomic analysis to identify genetic alterations that contribute to the progression to cervical SCC 5–8. These analyses have involved approaches such as array comparative genomic hybridization (aCGH), single nucleotide polymorphism (SNP) array, gene expression profiling, and fluorescence *in situ* hybridization (FISH). One of the most common genomic imbalances in cervical SCC is copy number gain and amplification of chromosome 5p, which occurs in up to half of advanced-stage cervical SCCs 5,9–11. Moreover, 5p gain is frequently seen in carcinomas at other anatomical sites including the head and neck 12, lung 13, and vulva 14, suggesting that it may be of broad relevance in oncogenesis. Interestingly, in the W12 *in vitro* system, which accurately models HPV16-associated cervical squamous carcinogenesis, 5p gain was rapidly selected over ∼15–20 population doublings and was associated with acquisition of the ability to invade collagen in organotypic tissue culture 15,16.

In order to identify driver oncogenes of importance in cervical squamous carcinogenesis, our group first performed aCGH in a set of 46 cervical SCC samples to identify regions of copy number gain that also showed amplification 5. The three most commonly occurring regions were all on 5p. We subsequently used gene expression analysis to identify candidate driver genes for which mRNA expression levels were significantly associated with gene copy number 5,17. Among these genes, the oncostatin M receptor (*OSMR*) and the microRNA processor *Drosha* were functionally validated and are likely to contribute to the selection of 5p gain in cervical carcinogenesis 5,17–19. Further FISH analysis on a tissue microarray of 110 independent cervical SCC cases showed that OSMR was copy number gained in ∼60% of the samples. Of interest, OSMR was not gained and overexpressed in low-grade or high-grade SILs, suggesting that such changes are relatively late steps in cervical carcinogenesis 5. Other groups have confirmed that OSMR is copy number gained and overexpressed in independent datasets of cervical SCCs 11,20, although there have been no studies to date of cervical adenocarcinomas. Importantly, OSMR copy number was associated with decreased overall survival in cervical SCCs treated by radiotherapy, independently of tumour stage (*p* = 0.046) 5. While this finding reflects observations in breast carcinomas (see below), it was from a single set of cervical SCC samples and needs to be verified independently.

## Oncostatin M receptor in health and disease

OSMR is a member of the interleukin 6 (IL6) receptor family. It associates with gp130 to form the high affinity receptor for its major ligand, the cytokine oncostatin M (OSM), and is also able to bind IL31. Following OSM binding, OSMR signals mainly through the JAK/STAT pathway, but also activates MAPK/ERK and PI3K/AKT cascades, inducing the transcription of context-dependent target genes 21,22. OSM is a multifunctional cytokine produced mainly by leukocytes, such as T cells, macrophages, and neutrophils 22. In contrast, OSMR is expressed in a wide range of cells, including leukocytes, endothelial cells, hepatocytes, neurons, and some epithelial cells (eg from breast, skin, and lung) 22,23.

OSM–OSMR signalling has key roles in inflammation, haematopoiesis, and development, and is increasingly being recognized as an important contributor to cancer progression 22. OSMR is expressed by many tumour cell types, including sarcoma, melanoma, glioma, and some carcinoma cells (eg from breast and prostate) 22. In breast cancer, high OSMR expression correlated with shorter recurrence-free and overall survival (*n* = 321 cases) 24 and with chemotherapy resistance in oestrogen receptor-negative tumours (*n* = 114 cases) 25. In addition, levels of OSM were elevated in breast 26, hepatocellular 27, and prostate carcinomas (where OSM expression increased with Gleason grade 28). The main source of OSM in cancers seems to be the tumour stroma, primarily the tumour-associated macrophage component, suggesting the existence of paracrine signalling between tumour stroma and cancer cells 24,26,29. However, OSM production has also been observed in some carcinoma cells, including those from the breast and prostate, indicating the potential for additional autocrine signalling 26,28.

The role of OSM in cell proliferation seems to be context- and cell type-dependent, with pro-proliferative effects in some cells 30–32 and anti-proliferative effects in others 33–36. C-MYC has been suggested to act as a molecular determinant of cellular responses to OSM, as OSM-mediated growth arrest requires efficient down-regulation of C-MYC expression by STAT3. Therefore, the expression levels of C-MYC and its ability to be effectively suppressed will determine whether cells arrest or proliferate upon OSM treatment 29. OSM can also affect other hallmarks of cancer. For example, it induces cell detachment, anchorage-independent growth, migration, and invasion in breast cancer cells 26,29,37, and increases tumour growth and metastasis in *in vivo* xenograft models of prostate and breast cancer 26,38,39. The molecular mechanisms underlying OSM–OSMR effects in cancer cells generally remain poorly understood. Recent reports suggest that in breast cancer, OSM may promote epithelial–mesenchymal transition 25,26 and suppress oestrogen receptor-α expression 24. Some tumours, including lung adenocarcinomas and oesophageal SCCs, seem to express a truncated and soluble form of OSMR, which possibly operates as a decoy receptor for OSM 40–42.

## Functional significance of OSMR overexpression in cervical SCC

**Figure 1:**
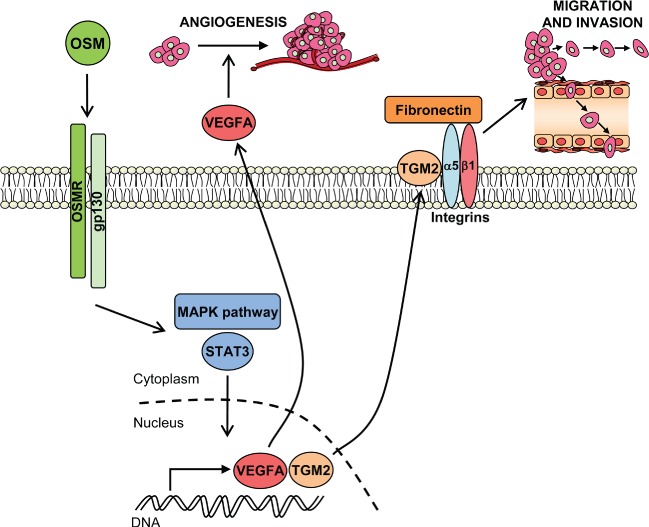
Known pro-malignant effects of OSMR in cervical SCC cells. Binding of OSM to the receptor subunits OSMR and gp130 activates STAT3 and MAPK pathways. This leads to the transcription of target genes, including vascular endothelial growth factor A (*VEGFA*) and transglutaminase 2 (*TGM2*). VEGFA is secreted by the cervical SCC cells, so activating angiogenesis. Cell membrane-associated TGM2 interacts physically with integrin-α5β1, acting as a co-receptor for fibronectin and inducing cell migration and invasion.

Our group has studied the biological basis of the association between OSMR overexpression and adverse clinical outcome in cervical SCC. In representative OSMR-overexpressing cervical SCC cell lines, OSM activated (ie phosphorylated) STAT3, p44/42 MAPK, and S6 ribosomal protein, effects that were reduced after OSMR depletion using RNA interference (Figure 1) 5. These observations were in agreement with data suggesting that STAT3 is the main STAT transcription factor activated by OSM–OSMR in transformed and non-transformed cells 22. We next studied the effects of OSM–OSMR interactions on the phenotype of cervical SCC cells by using complementary *in vitro* approaches including gene depletion and overexpression 18. By comparing cell lines that overexpressed OSMR with those showing no OSMR overexpression, we concluded that OSMR up-regulation conferred increased sensitivity to OSM, which induced a pro-malignant phenotype, via both direct and indirect effects.

The principal indirect effect of OSM–OSMR interactions in cervical SCC cells was to promote a pro-angiogenic phenotype. OSM induced a significant increase in VEGFA production in OSMR-overexpressing cells, while conditioned medium from OSM-stimulated cervical SCC cells induced angiogenesis in an endothelial–fibroblast co-culture model system 5,18. The latter effect was inhibited by depleting OSMR in the SCC cells and by pre-incubating the conditioned medium with a neutralizing antibody against VEGFA. Together, these findings showed that VEGFA was the principal mediator of angiogenesis induction by the OSMR-overexpressing cervical SCC cells (Figure 1) 18.

The direct effects of OSMR overexpression on cervical SCC cells were to increase cell migration (measured in a wound healing assay) and invasiveness (measured using Matrigel Boyden chambers) (Figure 1). While these effects were induced by OSM and inhibited by OSMR depletion in OSMR-overexpressing cells, OSMR depletion in the absence of exogenous OSM also decreased cell migration and invasiveness 18. This suggested endogenous OSM production by cervical SCC cells, which we subsequently confirmed using PCR (unpublished data). Consistent with its effects on increasing cell motility, OSM reduced the colony-forming efficiency (CFE) of OSMR-overexpressing cervical SCC cells, while OSMR depletion produced a significant increase in CFE 18. Exogenous OSM and/or OSMR depletion had no effect on cervical SCC cell proliferation 18, supporting the evidence that OSM effects on proliferation are cell type- and context-dependent 22.

## Molecular mechanisms for pro-malignant effects of OSMR in cervical SCC

We used integrative gene expression profiling to identify potential mediators of the ligand-dependent phenotypic effects of OSMR overexpression in cervical SCC cells. We identified genes that were induced by OSM in cells that overexpressed OSMR but not in cells where OSMR was not overexpressed. The most significant gene ontology category groups for the differentially expressed genes included angiogenesis, cell motility/invasion, and signal transduction, consistent with the phenotype observed in OSMR-overexpressing cells after OSM treatment 18. Of particular interest were 15 genes that also showed an association with OSMR levels in a parallel analysis of 23 cervical SCC tissue samples 18. These genes were strong candidate mediators of OSM–OSMR effects in cervical SCC cells, as they showed consistent association with OSMR levels, both *in vitro* and *in vivo*.

The 15 genes included the pro-inflammatory cytokine interleukin 6 (*IL6*), which is a well-known STAT3 target and further activates STAT3 43, indicating a probable positive feedback loop in cervical SCC. Also included was hypoxia-inducible factor 2-alpha (*HIF2*α or *EPAS1*), which was induced by OSM in normoxic OSMR-overexpressing cells. This gene is an interesting candidate mediator of the pro-angiogenic effects of OSMR-overexpressing cells. A third gene of note was transglutaminase-2 (*TGM2*), also known as tissue transglutaminase, a multifunctional protein with both enzymatic (crosslinking) and non-enzymatic functions. These functions are closely related to the subcellular location of TGM2 and also depend on the patho-physiological context 44. TGM2 is overexpressed in a range of cancer types, where it is associated with metastasis and decreased patient survival 45. In a combined tissue sample and *in vitro* study, we showed that TGM2 was an important mediator of the ligand-dependent phenotypic effects of OSMR overexpression in cervical SCC cells (Figure 1) 46. TGM2 depletion in cervical SCC cells abrogated the increased migration and invasiveness induced by OSM, while its ectopic expression increased cell motility and invasion. TGM2 interacted physically with integrin-α5β1 and fibronectin in cervical SCC cells (Figure 1) and OSM treatment strengthened the interaction. Importantly, integrin-α5 (*ITGA5*) was also one of the 15 genes identified in our comparative microarray analysis of OSMR targets 18. Moreover, levels of members of the TGM2–integrin-α5β1–fibronectin pathway correlated with disease progression and with OSMR levels in cervical SCC tissue samples 46. Taken together, these results showed that an OSMR–TGM2–integrin-α5β1 pathway is of biological and clinical significance in cervical SCCs (Figure 1).

## Clinical significance of OSMR overexpression and future directions

The findings summarized in this review indicate that OSM–OSMR interactions are a promising candidate for therapeutic targeting in cervical SCC. Moreover, there is growing evidence that an anti-OSM–OSMR therapy could also be effective in other SCCs. By interrogating publicly available microarray datasets and using real-time qPCR, we found that OSMR is overexpressed in oral, larynx, vulvar, and skin SCC (unpublished observations) and that the TGM2–integrin-α5β1–fibronectin pathway downstream of OSMR activation is overexpressed in head and neck as well as cervical SCCs 46. There remains a requirement for further studies using clinical material to confirm the relationship between OSMR overexpression and neoplastic progression in new, independent sets of tissue from cervical SCCs, as well as carcinomas from other sites. For tumours in the latter group, it will be important to include an assessment of the relationship between OSMR levels and HPV status, which to date has not been possible using available datasets.

Taking advantage of the fact that OSMR is a cell surface receptor and OSM an extracellular ligand, a rational strategy to target OSM–OSMR interactions is antibody-based inhibition. This therapeutic approach has had a major impact on haematological malignancies and solid tumours (such as ERBB2-positive breast cancers or EGFR-positive colorectal carcinomas) via a range of beneficial effects, including growth arrest, induction of apoptosis, inhibition of angiogenesis, etc 47. Twelve antibodies have received approval in the USA for the treatment of various cancers and several others are currently being tested in clinical trials 47. Interestingly, humanized antibodies against OSM are showing considerable promise in treating rheumatoid arthritis, where they inhibit leukocyte migration and reduce inflammation 48. Our accumulated data 5,18,46 suggest that analogous benefits will also be obtained in cervical SCC (and potentially other SCCs where OSMR is overexpressed) by reducing tumour cell migration and invasion and inhibiting angiogenesis. As OSMR is overexpressed in cervical SCC cells compared with normal cells, the therapeutic window for using OSM–OSMR blocking antibodies is likely to be relatively wide.

*In vivo* studies using appropriate preclinical models are now required to test the benefits of targeting this functionally significant receptor pathway in cervical and other SCCs. In addition to well-studied cell line xenografts 49, it will also be of interest to use patient-derived xenografts (PDXs) from clinical samples of OSMR-overexpressing SCCs. PDXs are explants from patient-derived tumour tissue engrafted into immuno-compromised mice. They conserve original tumour characteristics such as global gene-expression patterns, mutational status, metastatic potential, drug responsiveness, and tumour architecture 50, and will therefore be patho-physiologically relevant models to test the anti-tumour effects of OSM–OSMR blockade.
